# Resolving signal drift in the wall‐mounted camera of the RGSC system

**DOI:** 10.1002/acm2.14291

**Published:** 2024-02-02

**Authors:** Jeonghoon Park, Justin D. Gagneur, Suzanne J. Chungbin, Yi Rong, Seng Boh Lim, Maria F. Chan

**Affiliations:** ^1^ Department of Medical Physics Memorial Sloan Kettering Cancer Center Basking Ridge New Jersey USA; ^2^ Department of Radiation Oncology Mayo Clinic Phoenix Arizona USA

**Keywords:** camera calibration, motion management, RGSC

## Abstract

**Purpose:**

To present a modified calibration method to reduce signal drift due to table sagging in Respiratory Gating for Scanner (RGSC) systems with a wall‐mounted camera.

**Materials and Methods:**

Approximately 70 kg of solid water phantoms were evenly distributed on the CT couch, mimicking the patient's weight. New calibration measurements were performed at 9 points at the combination of three lateral positions, the CT isocenter and ±10 cm laterally from the isocenter, and three longitudinal locations, the CT isocenter and ±30 cm or ±40 cm from the isocenter. The new calibration was tested in two hospitals.

**Results:**

Implementing the new weighed calibration method at the extended distance yielded improved results during the DIBH scan, reducing the drift to within 1 from 3 mm. The extended calibration positions exhibited similarly reduced drift in both hospitals, reinforcing the method's robustness and its potential applicability across all centers.

**Conclusion:**

This proposed solution aims to minimize the systematic error in radiation delivery for patients undergoing motion management with wall‐mounted camera RGSC systems, especially in conjunction with a bariatric CT couchtop.

## 1 INTRODUCTION

Intrafraction motion is a known issue in radiotherapy that becomes especially important with the advancements in precision radiotherapy. In the past few years, there has been a growing incorporation of respiratory motion management into standard clinical practice. This rise can be attributed to the development of practice guidelines and the outcomes of clinical trials.^1^, ^2^, ^3^, ^4^ In light of the evolution and advancements in respiratory motion management since the AAPM TG 76 report published in 2006,^5^ the AAPM has established TG 324. In a 2020 survey conducted by AAPM TG 324 with 527 respondents, the clinical practice of respiratory motion management in radiation oncology was quantified.^6^ The results demonstrated that 84% of the respondents utilized Deep Inspiration Breath Hold (DIBH) for left‐sided breast cancer, while 95% employed motion management techniques for thoracic and abdominal cancers. The survey also highlighted the prevalence of specific devices for guided DIBH in breast cancer patients, with Real‐time Position Management/Respiratory Gating for Scanners systems being the most commonly used (41% of 262 respondents), followed by the AlignRT/Optical Surface Monitoring System (38%).

RGSC (Respiratory Gating for Scanners, Varian Medical Systems, Palo Alto, CA) is a motion management solution for CT simulation. RGSC is distinguished from its precedent RPM (Real‐time Position Management, Varian Medical Systems, Palo Alta, CA) with a new reflector block of four infrared markers, cameras, software, data processing algorithms, and integration with the ARIA oncology information system (Varian Medical Systems, Palo Alta, CA). Extensive testing has affirmed the stability of RGSC systems, demonstrating their ability to achieve superior dynamic treatment precision.^7^, ^8^ Most RGSC cameras were installed as “couch‐mounted” to provide a constant distance between the camera and the block during the CT scan and it was seamlessly integrated with various CT scanners. However, new Siemens go.Open Pro CT does not offer the option for a couch‐mounted camera anymore due to the maximum table sagging of 8 mm and the subsequent impact on RGSC trace as alerted by Varian.^9^ Consequently, users must opt for the ceiling or wall‐mounted configuration, leading to a significant clinical challenge.

Recently, Mayo Clinic in Phoenix and MSKCC in Basking Ridge installed Siemens go.Open Pro CT scanners with a bariatric couch (weight limit 307 kg) and a wall‐mounted RGSC camera. A rigorous commissioning test was conducted for the new CT scanner, following the recommendations of AAPM TG 66^10^ and institutional guidelines, including the RGSC system v2.0 integrations and 4DCT commissioning using a commercial motion phantom. However, both hospitals experienced noticeable baseline drifts on the RGSC trace during the DIBH and 4D scans as previously reported to have a baseline drift of 1.83 up to 6 mm.^11^ This issue, which has persisted for a period, results in inaccurate treatment if not managed diligently by clinical users. This technical report urgently addresses a prolonged concern regarding the drifting issue of wall/ceiling‐mounted RGSC cameras specifically for Siemens go.Open Pro CT scanners. The new calibration method is presented to assist the clinical physics community in effectively tackling this issue.

## 2 METHODS

### 2.1 Issues with the current calibration

**FIGURE 1:**
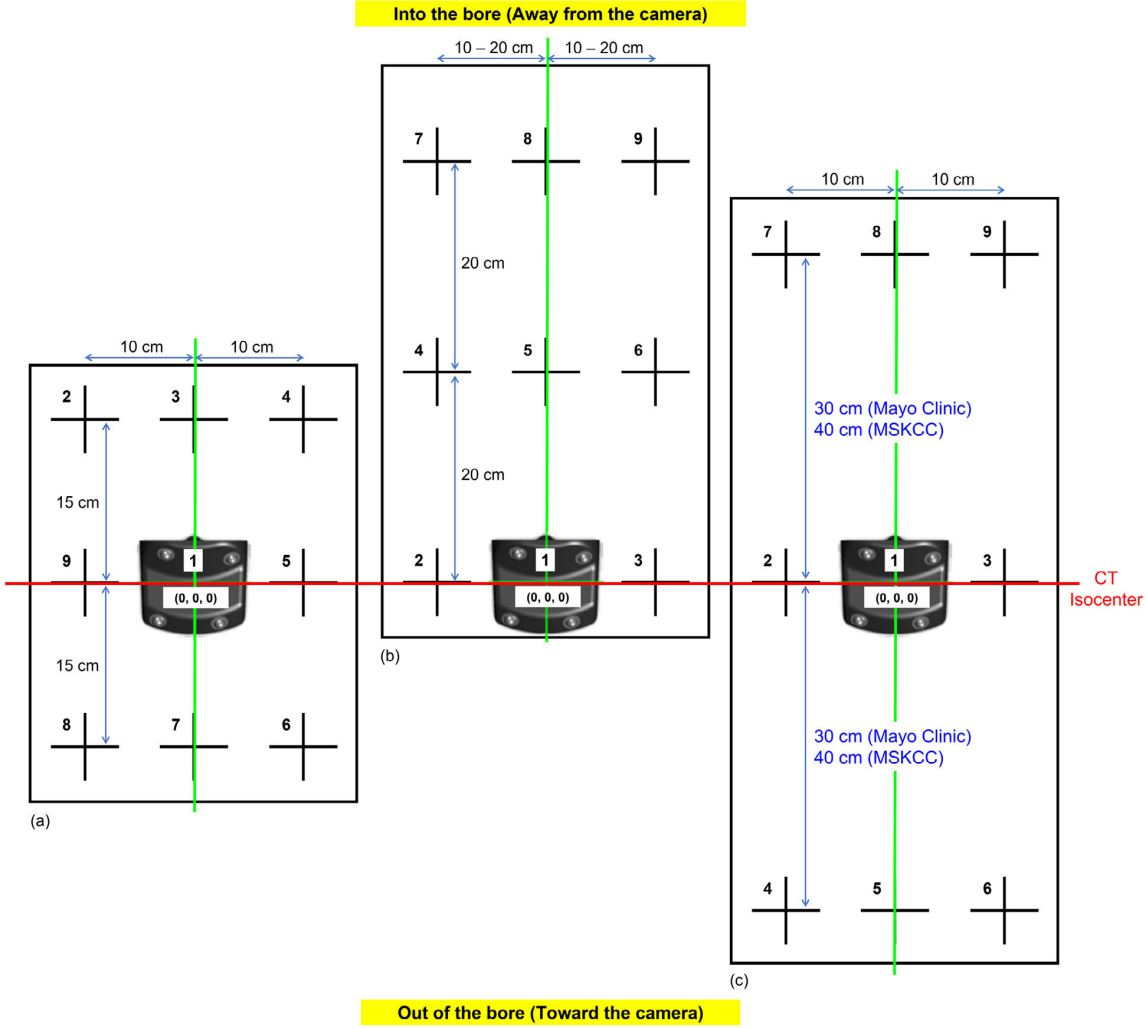
Comparison of the current and proposed calibration method (a) Varian standard calibration; (b) Varian alternative calibration with weight; (c) Proposed extended calibration with weight.

RGSC system requires periodic camera calibration and verification, especially when employing wall‐mounted or ceiling‐mounted cameras. This calibration process establishes a correlation between the longitudinal position of the reflector block (i.e., couch) and the shape of the IR marker because the distance varies between the camera and the marker block during the CT scan. The vendor's standard calibration is conducted on a 20 × 30 cm^2^ calibration plate with 9 equidistance points, as shown in Figure 1a. Following the detection of baseline drift, an independent verification of the reflector block's position was conducted at various vertical and longitudinal couch positions. Further investigation uncovered that the reflector block position was accurate only within or inferior to the 30 cm calibration length, especially when performed without a patient. The subsequent focused analysis enabled us to identify the factors contributing to the drift, particularly when a patient‐equivalent load is present on the couch.

### 2.2 Proposed calibration

An alternative calibration method is advised in the presence of baseline drift, as indicated by the vendor's reference.^12^ The method involves utilizing extended distances at least 40 cm superior to the CT isocenter, as shown in Figure 1b, achieved by evenly distributing a 70 kg weight on the couch. However, even with this alternative approach, a similar drift was still observed if the reflector block moved beyond the calibrated region, especially inferior to the CT isocenter, an area not covered by the calibration. Following the communication with the vendor, it was clarified that a 9‐point calibration is used to determine the vertical position of the reflector block across various couch positions, and the sequence of acquiring calibration points does not impact the outcome. Consequently, the lack of a corresponding IR marker shape beyond the calibrated region contributes to signal drift in the alternative calibration, in addition to variations in couch sagging with/without a patient's presence. Through meticulous efforts, we developed a calibration resolution covering the extended regions superiorly/inferiorly, as shown in Figure 1c.

First, about 70 kg of solid water phantoms were evenly distributed on the couch, mimicking the patient's weight, following the vendor's recommendations. Then, the reflector block was placed at the reference position #1, for example, the CT isocenter, of the white calibration plate to begin the calibration procedure. Subsequent calibrations were performed at 10 cm left/right of the first reference position, as depicted in Figure 1c. The couch was moved 40 cm (30 cm for Mayo Clinic) inferiorly from the isocenter to continue calibrations at points 4−6. Finally, the couch was moved 40 cm superiorly (30 cm for Mayo Clinic) to complete the remaining calibrations at points 7−9. Note that the reflector block could be placed at the approximate locations for calibration positions #2 to #9. Following this, a new DIBH scan was performed with the Varian motion phantom to validate the RGSC trace. To summarize, new 9‐point calibration measurements were performed: at three lateral positions, CT isocenter and ±10 cm laterally, at three longitudinal locations from the CT isocenter, ±30 cm or ±40 cm. This calibration is expected to be performed monthly or when the verification fails.

## 3 RESULTS

**FIGURE 2:**
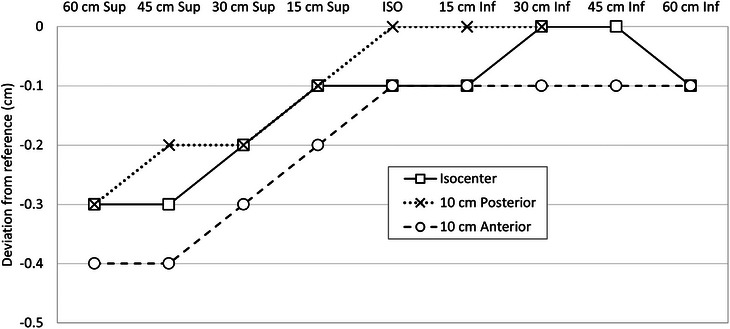
Verification of the vertical position of the reflector block at different longitudinal positions. The vertical positions were measured in the verification mode of the RGSC system after repositioning the longitudinal couch position using the CT console.

**FIGURE 3:**
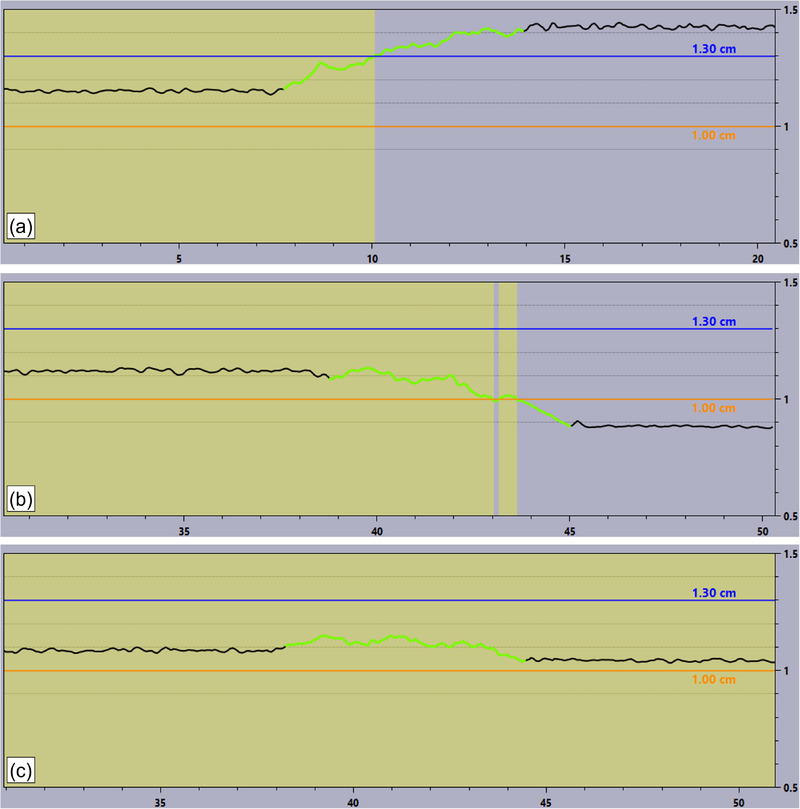
Comparison of DIBH traces with 70 kg weight for different camera calibrations. Green trace represents the beam on periods in the CT (a) Varian standard calibration; (b) Varian alternative calibration with weight; (c) Proposed extended distance calibration with weight.

Under stationary and without additional load conditions, all RGSC commissioning tests, including 4DCT and DIBH, met the specified technical standards and tolerances (≤2 mm).^7^, ^10^, ^12^ However, during stationary position verification after the standard calibration, approximately 60 cm superior to the CT isocenter, a vertical discrepancy of 3 mm was observed compared to the reference position. Conversely, within 60 cm inferiorly to the isocenter, the values matched the calibrated measurements precisely, as illustrated in Figure 2. This is deemed to be assisted by the rigid couch base structure holding the inferior part of it while its superior part is floating. This variability introduced more complexity to the DIBH scans. The trace exhibited a downward drift as the couch moved away from the camera and an upward drift as the couch moved approached the camera, as depicted in Figure 3a. This drift could potentially cause the DIBH trace to exceed the predetermined thresholds during the scan, set at 5 mm for breast cases and 3 mm for others in MSKCC. In addition, trace drifts of approximately 4−6 mm were also observed during patient scans due to the additional couch sagging. The same phenomenon was observed in other wall‐mounted RGSC installations with CT scanners from various vendors. Siemens go.Open Pro CT is most impacted, particularly due to the bariatric couchtop, as explained in the introduction section. The DIBH motion of the patient was found to be accurate at any location as long as the breathing trace was rebaselined at the scan start position. However, the user must adhere to the initial trace for accurate DIBH thresholds because the rest of the traces are biased by the observed drift when the standard calibration is applied. Moreover, the use of VCD (Visual Coaching Device, Varian Medical Systems, Palo Alto, CA) is still cautioned in these circumstances because the biased signal is transmitted to the VCD in real‐time.^9^

DIBH scans were performed using the breast CT protocol with 70 kg weight in MSKCC for three different camera calibrations as shown in Figure 3. The scan length was 50 cm and the couch moved toward the camera. Implementing the new weighed calibration method at the extended distance yielded improved results during the DIBH scan, reducing the drift to within 1 mm, as illustrated in Figure 3c. Both extended calibration positions, at ±30 cm or ±40 cm, exhibited similarly reduced drift in both hospitals, reinforcing the method's robustness and its potential applicability across all centers. However, it is noteworthy that the new calibration method may still exhibit a larger residual drift if the patient's weight significantly differs from the calibration weight. The direction of the residual drift is contingent upon the patient's weight relative to calibration weight. A lighter patient can lead to an under‐compensation of couch‐sagging, causing the trace to drift downward when the couch moves toward the camera. Conversely, the trace drifts in the opposite direction for a heavier patient. Despite this weight‐dependent variability, the extended weighted calibration method greatly advances mitigating drift during DIBH scans.

## 4 DISCUSSION AND CONCLUSIONS

A new RGSC camera calibration method was developed to mitigate the baseline drift in the bariatric CT simulator couch. Historically, most RGSC cameras were installed as “couch‐mounted” seamlessly integrated with a range of CT scanners from the authors’ experiences, including models from Philips and GE. However, the baseline drifting was a long‐standing issue in non‐couch‐mounted RGSC cameras.^11^ Despite the extensive research efforts to identify an acceptable calibration procedure and clinical resolution, these efforts did not manage to bring the drift down to the level observed in the couch‐mounted camera. The implications of baseline drift in DIBH scans are profound; it can lead to incorrect breathing thresholds and, if not rectified by vigilant user intervention, potentially result in inaccurate treatments. Furthermore, in the context of 4D scans, drift can lead to inaccurate binning results, especially when amplitude‐based binning is used, while the phase‐based 4D CT has minimum impact under stationary acquisition and binning. In light of these challenges, the authors urgently propose a new camera calibration method for wall‐mounted RGSC cameras, utilizing weight calibration in the extended calibration region. This approach has demonstrated the ability to reduce baseline drift to within 1 mm. Significantly, the proposed calibration method is also applicable to ceiling‐mounted cameras. Moreover, it is advisable for users to mount the camera adhering to the vendor's recommended range of 350–500 cm from the CT isocenter and the optimal projection angle. The optimal distance between the camera and reflector block is approximately 430−500 cm, consistent across both hospitals.

Initial implementation of our new method in a patient breathing trace acquisition has shown promising results; however, certain areas necessitate further refinement as we continue to advance this crucial technology. Daily camera verification posed challenges without proper weight, resulting in a deviation of about 2 mm, which still falls below the acceptable tolerance of 5 mm. This situation leaves the users to either conduct the verification on the empty couch or employ any motion phantom for regular checks. The observed drift directions and magnitudes depended on the patient's weight discrepancy from the calibration weight. The overall deviation was maintained at within 1 mm, but potential discrepancies were recognized for patients significantly lighter or heavier than the calibration weight (70 kg). Multiple weight calibration options need to be implemented in the next RGSC application to allow the user selection per the patient's weight. This extended calibration applies to all scanning modes available in the RGSC system, not limited to “Breath‐hold.” In addition, the proposed method has not been endorsed by the manufacturer, and a user who decides to implement the proposed calibration needs to carefully study the clinical performance of the device after changing the calibration. A recent study conducted by Liu et al. compared the baseline drifts when using three reflector blocks versus using a single reflector block for calibrating of wall‐mounted RGSC camera.^13^ Their three‐block method provides a new 9‐point calibration scheme from a similar background to this study and results in a residual drift of 0.2 mm on an empty couch. Nevertheless, the most notable distinction between the two methods lies in our calibration incorporating a weight factor (70 kg). We contend that the inclusion of weight is crucial to simulate the patient's presence on the couch, thereby accounting for any sagging, that is, AP motion, of the couch during CT scanning. Please note that the daily, monthly, and annual QA procedures for the RGSC still adhere to the recommendations set forth by the authors’ institutions. This study exclusively focuses on enhancing the monthly QA of the wall‐mounted camera within the RGSC.

Despite the outstanding results of the proposed method, a couch‐mounted camera system is deemed to be a definite solution to avoid the cumbersome camera calibration and to remove residual errors, with the prospect of upgrading the current bariatric couch to a more rigid one when it becomes available to the new CT simulator. Additionally, a product improvement request was made to the vendor on the creation of a standardized 70 kg phantom for therapists and physicists, facilitating daily verification and monthly RGSC calibration tailored to mitigate this drifting issue. This solution will significantly reduce the systematic error in the radiation delivery for patients under motion management with such systems. To safeguard patient safety and treatment accuracy, it is imperative that the RGSC system manufacturer promptly initiates an urgent user notice and provides appropriate technical advisories. Patient safety and treatment accuracy is paramount to ensure optimal outcomes.

## AUTHOR CONTRIBUTIONS

All authors significantly contributed to the study. Jeonghoon Park performed CT and RGSC commissioning in MSKCC. Justin D. Gagneur performed RGSC commissioning and a new calibration procedure. Suzanne J. Chungbin performed the 4D CT commissioning test and analysis. Yi Rong led new CT commissioning as a project leader at Mayo Clinic. Seng Boh Lim contributed to the new CT commissioning as a Director of Radiation Dosimetry Core. Maria F. Chan wrote a draft of the manuscript and led the CT commissioning as a project leader at MSKCC. All authors reviewed the manuscript and approved it.

## CONFLICT OF INTEREST STATEMENT

The authors declare no conflicts of interest.
